# The Landscape of Obesity Education Worldwide — Are We Doing Enough? Scoping Review of Content of Obesity Educational Interventions in Medical Schools and Residency Programs

**DOI:** 10.1007/s11695-024-07654-y

**Published:** 2025-03-03

**Authors:** Wahiba Elhag, Walid El Ansari

**Affiliations:** 1 Dept. of Bariatric Surgery and Bariatric Medicine, Hamad Medical Corporation, Doha, Qatar; 2 Weill Cornell Medicine – Qatar, Doha, Qatar; 3 College of Medicine, Ajman University, Ajman, UAE; 4 Dept of Surgery, Hamad Medical Corporation, Doha, Qatar

**Keywords:** Obesity education, Medical school, Residency programs, Content, Scoping review

## Abstract

No study appraised the curricular content of published obesity education interventions (OEI) delivered to medical students, residents, or fellows. This scoping review identified and described the curricular content of published OEI, examined the specific obesity-related domains addressed, and proposed potential directions for future intervention development. We searched PubMed and Web of Science databases. Original articles on OEI delivered to medical students, residents, or fellows were included. Data extraction was guided by the Obesity Medicine Education Collaborative’s framework and the Accreditation Council for Graduate Medical Education-International (ACGME-I) competencies framework. The curricular content of OEI was categorized and visually mapped to identify trends and gaps. A total of 490 studies were identified, and 60 were included. Conceptually, twelve obesity domains emerged: epidemiology, health policy, prevention; basic sciences; nutrition; physical activity; behavioral aspects; counseling; pharmacotherapy; metabolic/bariatric surgery (MBS); weight stigma; ethics/professionalism; health literacy; and practice guidelines. Geographically, most OEI (88.3%) were implemented in North America. Three-quarters of OEI were for medical students, less OEI were for residents, and none for fellows. Content-wise, the OEI for medical students and for residents exhibited strikingly limited coverage of all obesity domains, although those for residency programs had more limited coverage than those for medical schools. Across medical schools, the most common domains addressed were basic sciences (48.3%), counseling, and nutrition (45% each); the least included were pharmacotherapy (13.3%), ethics/professionalism (3.3%), and health literacy (1.6%). For residency programs, the most common domains addressed were basic sciences and counseling (21.6% for each); the least were pharmacotherapy and MBS (6.6% each), weight stigma (5%), ethics/professionalism (3.3%) and health literacy (1.6%). Many critical domains of obesity remain inadequately addressed in the education provided in medical schools and residency programs. Future OEI should incorporate these to equip future physicians with knowledge, skills, and attitudes to care for patients with obesity.

**Supplementary Information:**

The online version contains supplementary material available at 10.1007/s11695-024-07654-y.

## Introduction

The prevalence of obesity has nearly tripled since 1975, signifying a major health concern globally [1]. Obesity is a multifactorial chronic relapsing disease with complex pathogenesis, and high comorbidity, disability, and mortality [2–4]. Given its clinical, psychological, and functional complications, obesity management requires multidisciplinary approaches combining dietary, physical activity, behavioral, pharmacological, or surgical interventions tailored to the patient’s needs. Hence, physicians play a vital role in the diagnosis, counseling, and treatment of patients with obesity (PWO). However, despite this, the diagnosis and management of obesity by physicians remain suboptimal [5, 6].

One of the recognized barriers to effective management of obesity is the suboptimal education of physicians during medical school and residency [7]. Such shortcomings include knowledge deficits about obesity-related patient services and counseling as well as inadequate evidence-based treatment modalities, nutritional interventions, and behavioral modification strategies [8, 9]. The suboptimal education also include weight stigma and negative beliefs and attitudes toward PWO [8, 9]. Indeed, physicians themselves believe they have been inadequately trained to deal with obesity issues [10]. This highlights the importance of comprehensive obesity content in the educational syllabi of medical students and residents.

It is important that physicians are competent and knowledgeable to be able to provide optimal care to PWO. However, despite the pressing need for comprehensive obesity education that spans undergraduate and graduate medical education, progress has been slow. Reasons for the insufficient obesity education and training include few qualified faculty, limited time, crowded curricula, and lack of recognition of obesity by primary or specialty boards [11–13]. Hence, medical schools as well as residency and fellowship programs must ensure that the education they provide for future physicians is tightly aligned with the skills required to combat the obesity epidemic and provide optimal care for PWO. This requires comprehensive curricula with content that addresses key obesity domains including knowledge, skills, behavior, and attitude to ensure that future physicians are well equipped to recognize, diagnose, and effectively manage obesity in their clinical practice and contribute to quality care and better patient outcomes.

Despite the importance of obesity education, the literature reveals knowledge gaps. To date, no reviews undertook comprehensive appraisals of the content of published obesity education interventions (OEI) designed and delivered to medical students, residents, or fellows. The four reviews that endeavored to undertake this task had limitations. For instance, a review exploring obesity education for medical students, residents, and fellows worldwide focused on the design and outcomes of the OEI, with no thorough assessment of their content, examining a limited time span by reviewing studies from 2005 to 2018 only [14]. Two other reviews addressed the effectiveness of OEI for medical students in facilitating lifestyle changes [15] or management of PWO [16], although both fell short of any in-depth “dissection” of the content of obesity domains addressed by these OEI. A fourth review examined OEI for medical students to identify gaps, presenting a brief table of samples of suggested curricular elements, but no detailed scrutiny of the curricular content of the included OEI [17]. In summary, some of these reviews were outdated or limited to the USA, and all did not conduct in-depth assessments of the OEIs’ content in terms of the obesity-related domains they addressed. These considerations acted as the drivers for the current review.

The current review bridges these gaps. Our primary objectives were to (a) identify published OEI designed and delivered to medical students, residents, or fellows; (b) extract detailed descriptions of the aims, goals, and curricular content of each OEI; (c) based on the curricular content of each OEI, determine the obesity domains it addresses; (d) map the specific domains examined by the OEIs vis-a-vis each other to identify trends and/or gaps; and (e) based on the identified gaps, suggest potential ways forward. To the best of our knowledge, this is the first study to undertake this task and the findings would be important for practitioners, educators, deans of medical schools, residency program directors, educational and curricular quality managers, policymakers, and others.

**Fig. 1:**
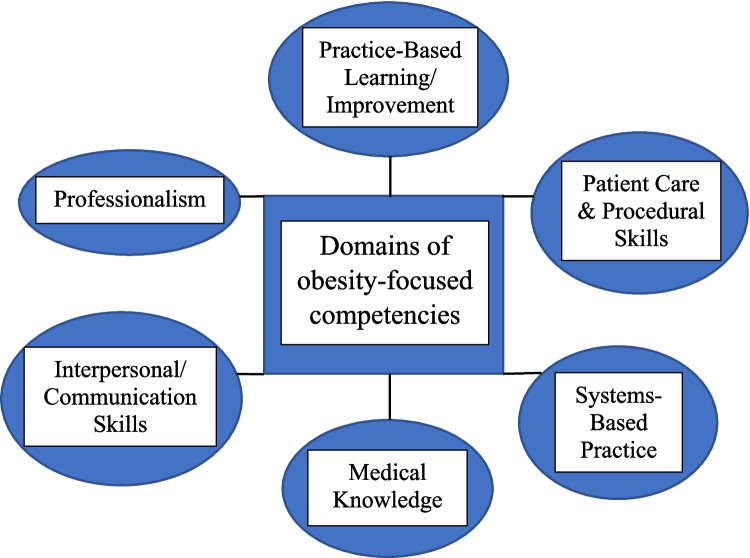
Framework of domains of obesity focused competencies guiding the current review (Adopted from [12, 18])

The current review was guided by a conceptual framework of the Obesity Medicine Education Collaborative (OMEC) framework (6 core domains, 32 associated competencies) [12], and the Accreditation Council for Graduate Medical Education-International (ACGME-I) competencies (six core competencies) [18]. These served as guidance for categorizing and analyzing the content of each OEI (Fig. 1).

## Methods

### Scoping Review

The intention of a scoping review is not to identify and account for every published information on the topic. Rather, the goal is broader, to interrogate the literature, uncover important aspects of the topic, unearth potential gaps, illustrate key examples, and synthesize research evidence, especially when the subject matter has not been reviewed in depth or is complex [19–21]. Thus, the scoping review was selected to appraise the content of OEIs in medical schools and residency and fellowship programs worldwide. It was conducted in accordance with the Preferred Reporting Items for Systematic Reviews and Meta-Analyses extension for scoping reviews (PRISMA-ScR) [22] and Arksey and O’Malleys’ six-step framework [23], ensuring and presenting procedural and methodological rigor and transparency [24, 25]. The six-steps are detailed below.

### Research Questions

The present review systematically scoped the literature to address four key questions related to each identified OEIs delivered to medical students, residents, or fellows, including (1) what are the published OEI designed and delivered to medical students, residents or fellows, and where are their locations?; (2) what are the goals of the OEI?; (3) what are the details of their curricular content?; and (4) based on the emerging findings, what are the gaps? The review sought to propose potential strategies to address such gaps, offering a way forward to improve obesity education.

### Identifying Relevant Studies

#### Information Sources

We searched PubMed and Web of Science electronic databases for published articles of all types related to OEIs.

#### Keywords and Search Terms

We included keywords and search terms such as “obesity education AND medical students,” “obesity education AND resident,” and “obesity education AND fellowship,” as well as their variations, e.g., “residency” and “residency program.” Supplementary Table 1 details the terms used within the search strategy. As the retrieved literature uncovered more features about OEI for medical students or residents, supplementary searches were formulated and conducted to obtain the literature related to the uncovered features.

### Study Selection

Inclusion criteria comprised original studies published in English between January 1, 1980, and April 1, 2024, focusing on OEIs for medical students or post-medical school trainees. Following the recommendations of the Center for Reviews and Dissemination for undertaking health care reviews, we determined our inclusion criteria based on PICOS configuration (population, interventions, comparators, outcomes, study design). The inclusion and exclusion criteria employed are depicted in Table 1. Included studies had to have sufficient description of the OEI content and its characteristics. Results were reported in accordance with the PRISMA guidelines [26].

**Table 1:** Inclusion and exclusion criteria for obesity educational interventions

Category	Inclusion criteria	Exclusion criteria
Population	Medical school students; medical trainees post medical school (residents, fellows); other participant groups (e.g., dieticians, nurses) may feature within the greater sample of medical students or trainees	Studies comprising only students or trainees of other health care professions (e.g., dieticians, nurses)
Intervention/s		
Nature	Must include educational intervention	No educational intervention: e.g., survey of view or opinions of learners or educators without intervention
Content	Educational intervention/s with explicit or implicit obesity or obesity-related content	Educational intervention/s with no explicit or implicit obesity or obesity-related content
Target	Educational intervention/s to improve patients’ health with or without improving learners’ health	Educational intervention/s to improve learners’ health only, with no explicit link to improving patients’ health
Comparator/s	Studies with or without control/comparison groups	Not applicable
Outcomes	Content characteristics described in sufficient detail, e.g., teaching and learning methods, health professions involved, duration, year or medical school or residency program, clerkship, etc	Studies without content characteristics reported
Study design/characteristics	All designs except reviews; English language; published 1980–2024; published/conducted in any country	Any type of reviews; studies published in languages other than English

### Charting the Data

The data extracted consisted of items relevant to the four research questions being examined. Together, the authors read each study comprehensively. Each study was content-analyzed to identify, highlight, and extract all obesity-related material relevant to the curricular content of each OEI into a spreadsheet. Both authors thoroughly re-examined each of the included studies, and attention was invested not to miss any curricular content information of any OEI. The extracted material was systematically categorized and organized into domains guided by the framework comprising OMEC and the ACGME-I core competencies. This process was repeated with each OEI thus ensuring comprehensive coverage and minimizing the risk of oversight. The findings were then summarized and presented as they relate to the review questions and objectives.

### Collating, Summarizing, and Reporting the Results

The review team gathered, organized, summarized, and presented the findings as they relate to the review questions and objectives and reported them below. Based on the emergent findings, we mapped the potential gaps that, if addressed, could offer valuable opportunities for advancing the field.

### Consultation Exercise

Two experts in bariatric medicine reviewed the findings to advise on and corroborate the findings of the review, and to suggest any important domains that could have been missed by the authors.

**Fig. 2:**
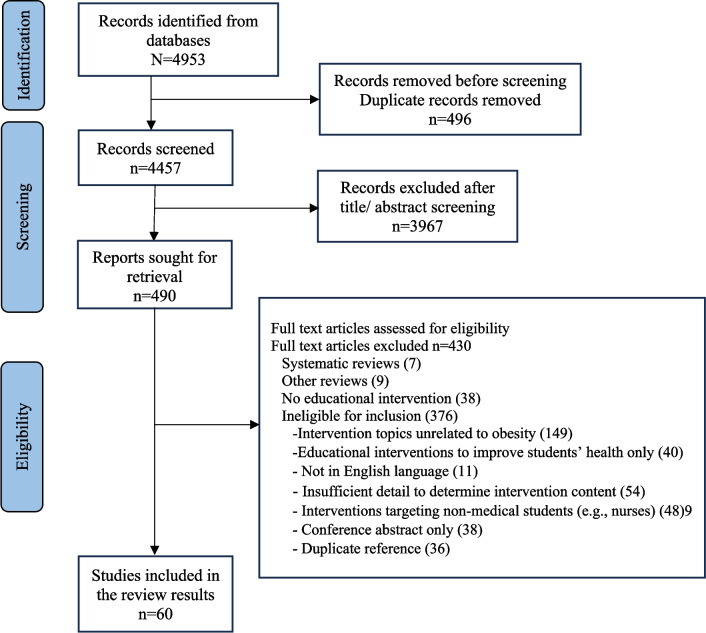
PRISMA flowchart of included studies

The searches returned an initial 4953 records. After the exclusion of duplicate records and screening of titles, 490 studies remained. Figure 2 shows the PRISMA flowchart of the included studies. A total of 60 articles were included in the current review.

## Results

### General Characteristics of Studies

**Fig. 3:**
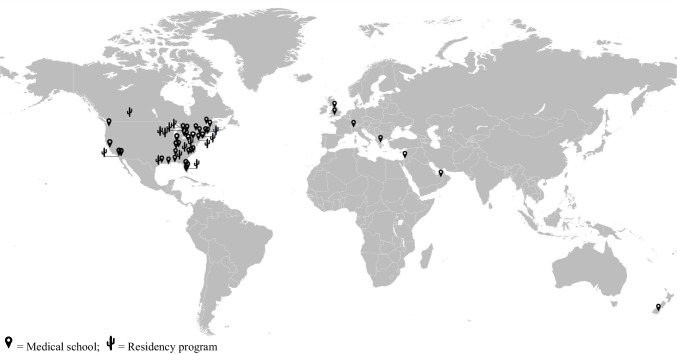
Geographical locations of obesity education interventions

Sixty studies were included [11, 27–85]. Geographically, most of these were from North America (88.3%), with fewer studies from six other countries (Table 2 and Fig. 3). In terms of learners, three-quarters of the OEI was aimed at medical students, with less OEI for residents (25%), and none for fellows. There was an increase in OEI during the last 10 years compared to the previous 30 years, with more than half the OEI published during the last decade.

**Table 2:** General characteristics of the obesity educational interventions (*N* = 60)

Characteristic	*N* (%)
Country	
North America	54 (88.5)
Other^*a*^	7 (11.5)
Target audience	
Medical students	45 (75)
Residency program	15 (25)
Year of publication^*b*^	
1982–2013	26 (42.6)
2014–present	35 (57.3)

^*a*^Includes UK, Switzerland, Greece, Turkey, Israel, UAE, and New Zealand

^*b*^2013 used as a cutoff, as it is the date when the American Medical Association (AMA) voted to recognize obesity as a disease state requiring treatment and prevention effort

### Goals, Content, and Other Characteristics of Included Studies

Tables 3 and 4 depict the obesity-related domain content of the 60 identified OEI in medical schools and residency programs worldwide. These domains were multiple and diverse and covered many aspects of obesity. Conceptually, these aspects were categorized into twelve obesity-related domains: epidemiology, health policy, prevention; basic sciences; nutrition; physical activity; behavioral aspects; counseling; pharmacotherapy; metabolic and bariatric surgery (MBS), weight stigma; ethics/professionalism; health literacy; and practice guidelines.

**Table 3:** Goals and content of obesity education interventions in medical schools worldwide

Study	Intervention goal/s	Content
Dengerink (1982) USA [27]	Incorporate direct practice into behavioral science	Obesity and behavioral intervention; treatment of obesity
Wiese (1992) USA [28]	Modify stigma toward PWO	Genetic causes of obesity, modify obesity stigma
Rourke (1999) USA [29]	Value of using total body composition in accurate diagnosis of obesity	Elective nutrition course: rationale/description of using 6 body composition techniques in obesity diagnosis/treatment; compare MS’s assessment of obesity using BIA and visual assessment, discuss obesity impact, diagnosis, treatment
Hodgson (2000) USA [30]	Increase learning and retention of nutritional concepts/skills	Longitudinal integrated curriculum: biochemistry, nutrition, laboratory course, pathophysiology, diet-assessment
Banasiak (2001) USA [31]	Enhance obesity knowledge	Obesity clinical clerkship & BS clerkship: obesity etiology, environmental/behavioral factors, medical risks and complications of obesity, diagnosis and treatment modalities; efficacy of MBS
Buchowski (2002) USA [32]	Enhance nutrition knowledge	Obligatory nutrition course: nutritional anemias, diabetes & weight management including epidemiology, biochemical, clinical, preventive, therapeutic perspectives of nutrition
Carson (2002) USA [33]	Cardiovascular nutrition module to improve knowledge, attitudes, self-efficacy and patient care	During the ambulatory care rotation: integrated cardiovascular nutrition module on nutrition therapy for patients with type 2 diabetes, hypertension, and hyperlipidemia. Focus is on lifestyle modifications, including healthy eating, weight reduction, and exercise
Carson (2003) USA [34]	Improve identification and treatment of metabolic syndrome	Information on metabolic syndrome; BMI/WC measurement; application of nutrition principles in assessment, treatment, referral of patients with elevated lipids, hypertension, and obesity; review obesity guidelines
Conroy (2004) USA [35]	Improve confidence about addressing patients’ diet, PA, and MS own health habits	Preventive medicine and clinical nutrition course: screening, immunization, counseling, chemoprophylaxis, nutrition in chronic diseases, dietary assessment/recommendations, PA, practice guidelines
Poirier (2004) USA [36]	Improve knowledge/confidence to counsel patients about behavior change; physician–patient communication skills	Behavior change counseling course: MI principles, reflective listening, assess readiness for change, effective interviewing/counseling skills, reducing resistance, exploring ambivalence, medication compliance
Endevelt (2006) Israel [37]	Familiarize with health risks of obesity, treatment strategies, dietary assessment and recommendations	Nutritional workshop: nutrition in prevention & treatment of disease; dietary assessment; nutrition recommendations; obesity epidemiology, pathology, health risks in adults/children, treatments, lifestyle changes, pharmacotherapy, MBS
Rodríguez (2006) USA [38]	Skills to define health problem in community (obesity), participate in educational intervention to support lifestyle change for patients: healthy eating, increase PA	Community based primary care experience in FM clerkship: group visits by 3rd/4th y MS to teach patients lifestyle modification; MS select lifestyle topics, produce patient education material to be taught during their visits to community health centers
Barss (2008) UAE [39]	Teach health determinants, healthy personal lifestyle, transferable generic skills	Lifestyle and causality of health, disease, family and personal lifestyle history, home observation; nutrition, exercise, smoking; food hygiene; practical skills in interviewing & history taking
Bell (2008) USA [40]	Improve MI knowledge, skills, confidence to counsel patients for health behavior change	Counseling curriculum as part of ambulatory care block during core medicine clerkship: MI principles/techniques; stages of behavior change; strategies to help patients begin change process
Dayal (2008) USA [41]	Develop obesity-related nutrition assessment and intervention skills	During obstetrics gynecology/women’s health clerkship: weight-related health issues in pregnancy (gestational diabetes); midlife (chronic disease risk, metabolic syndrome); older age (medical/social problems). Nutrition assessment, tailored intervention based on age, health, psychosocial issues
Zoberi (2008) USA [42]	Increase knowledge of common behavioral health issues in primary health care and their interventions	Interactive medical/psychological curriculum in FM clerkship: behavioral health (anxiety, depression, obesity, low sexual desire), integrated care; obesity epidemiology, diet, PA, medications, MBS, behavioral change to lose weight and maintain it
Moser (2009) USA [43]	Training in behavior change techniques	Mandatory communication and behavioral medicine course during ambulatory clerkship: physician–patient interactions, mind–body interactions in health/disease; counseling strategies for children, adolescents, adults, medication adherence; social inequalities, health policy/economics. MS assess own health behavior and how it applies to patient care
Schroder (2010) New Zealand [44]	Understand addictive overeating as potential disorder, increase empathy/confidence managing such patients	Component of Addiction Medicine training: MS attended overeating meeting and report their experiences of the meeting
Roberts (2011) USA [45]	Teach interdisciplinary care of MBS patients to improve attitudes toward obesity and MBS. Appreciate personal, social, medical, economic impacts of obesity on individual	Pilot longitudinal MBS elective during clinical clerkships: focus on MBS patients; MS interact with patient/family; participate in preop evaluation, intra/postop care; endocrinology and epidemiology of MBS and obesity; beliefs/stereotypes about obesity, health care systems, quality improvement, patient safety
McAndrew (2012) USA [46]	Train MS to educate adolescents on effects of obesity, motivate them to implement small healthy changes	Urban and community health course, MS create education intervention for HS students: HS students self-assessed own obesity knowledge/health behaviors. MS gave presentation on nutrition, psychosocial/medical implications of obesity; HS students encouraged to commit to health behavior challenge
Miller (2012) USA [47]	Help MS, educators and the public to learn about weight management	Family/Community Medicine program: obesity epidemiology; BMI classification, energy balance; environmental impact; obesity health effects/cancer risk; body image beliefs/attitudes, obesity bias/stigma; diet/PA counseling; weight loss strategies, behavioral change, pharmacotherapy, MBS, treatment guidelines
Poustchi (2013) USA [48]	Reduce bias toward obese patients	During nutrition course and FM clerkship: weight bias in health care; obesity experts present video with simulations of difficult situations patients faces in care settings to induce empathy, provide strategies for bias-free practices
Schmidt (2013) USA [49]	Improve knowledge, attitudes and counseling skills	During FM Clerkship: review national guidelines; assess patient BMI, obtain diet/weight history, assist patient in setting SMART goals. For extra credit, review paper on evidence-based weight loss, complete personal weight management plan
Birkhead (2014) USA [50]	Improve clinical nutrition competency	Integrated elective nutrition/cooking skills curriculum: training on healthy breakfasts, meal planning; MS led cooking/nutrition classes and chronic disease management education for underserved communities, provided healthy eating recipes
Kushner (2014) USA [51]	Improve attitudes, beliefs about obesity, confidence in communication	During communication skills Unit: structured education intervention; MS read articles on communication, stigma, took weight history of SP, discussed perception of weight, and how it affected them socially & physically
Matharu (2014) USA [52]	Diminish obesity prejudice, improve explicit/implicit bias	Standard lecture intervention: lecture on medical management. Intervention: play-reading incorporated women’s narratives and their understanding of their weight in context of social discrimination
Brown (2015) USA [10]	Teach delivery of evidence-based obesity intervention and provide WM program to underserved community residents	Extracurricular service-learning course on evidence-based treatment of obesity: definition, rates, costs, etiology; treatments for low-income individuals; general physician AAMC competency domains
Lee (2015) Canada [53]	Motivate MS to learn about impact of obesity	Game-based learning module on impact of obesity, obesity knowledge, confidence in making recommendations to patients
Milford (2016) USA [54]	Improve attitudes, knowledge, skills confidence regarding healthy literacy and patient communication	Community-based learning experience to train MS as family mentors for low-health literacy learners focusing on obesity prevention, nutrition, PA for children, parents, staff; poverty statistics, health care access barriers, health literacy skills
Chisholm (2016) UK [55]	Prepare students to support PWO to make changes to eating and PA patterns	Obesity-management session on complex behavior in obesity, behavior change: barriers, problem solving, coping; environmental change; decision-making, stress management; habit formation; empowering change; goal achievement; social support. Tailoring advice to patients, positive/negative doctor-patient interactions
Pasarica (2016) USA [56]	Increase knowledge and confidence to instruct patients on evidence‐based lifestyle management	Obesity definition, prevalence, comorbidities, guidelines; critical appraisal of EBM/non-EBM resources of commercial weight-loss programs; develop personalized recommendation for patient who wish to use commercial weight-loss program
Wilechansky (2016) USA [57]	Provide practical knowledge and counseling skills related to obesity, diet, exercise	Lifestyle management module in PC clerkship: obesity statistics, classification, implications; assessment, management, counseling of PWO; dietitians’ role in lifestyle management, healthy food choices, PA recommendations, MI strategies
Gayer (2017) USA [58]	Increase obesity knowledge, positively influence attitudes toward PWO	Obesity epidemiology, pathogenesis, associated chronic diseases, metabolic factors; nutrition, PA; behavior change; pharmacologic/nonpharmacologic interventions; metabolic syndrome, guidelines
Hawa (2017) Canada [59]	Increase knowledge/skill to understand, manage binge-eating disorder	Web-based self-learning module on BED during psychiatry clerkship: assessment of patients (interviewing, treatment planning); BED contributing factors, comorbidities, pathophysiology, treatment
Pasarica (2017) USA [60]	Improve knowledge on obesity management guideline	Self-contained module in IM/FM clerkship on guidelines/EBM (obesity management, dietary, PA, healthy sleep, drinking, MBS, behavior change, MI, pharmacotherapy), debunking myths, 5A approach
Ryan (2017) USA [61]	Improve attitudes, beliefs, confidence, knowledge of PA promotion	PA promotion/counseling: PA types, benefits; counseling to prevent, manage, treat chronic disease; PA strategies to modify behavior; health communication in clinical settings: HP engage empathically with individuals of all shapes/sizes
Broad (2018) UK [62]	Improve understanding of nutrition and PH	Nutrition/PH elective: physiology of healthy diets, pathophysiology of poor diet, MI techniques for healthy eating, clinical management of malnourishment, PH aspects of nutrition and obesity, nutrition with focus on pediatrics
Geller (2018) USA [63]	Improve attitudes toward PWO through innovative ethics session	Ethics/professionalism session: obesity causes; video clips with ethics/professionalism themes (adult male, preteen girl with obesity); MS reflect on own weight biases, link negative obesity attitudes to unprofessional behavior
Cohen (2019) USA [64]	Improve verbal communication with PWO	Behavioral and genetic causes of obesity/overweight
Leedham-Green (2019) Greece [65]	Improve knowledge, skills attitude in treating PWO	During General Practice placement: MI, behavior change theory, guidelines for nutrition, PA; 5As; reflective learning
Ockene (2021) USA [66]	Build, practice, develop positive attitudes toward WMC, behavior changes counseling skills	MS randomized to multimodal weight management education (theoretical constructs at individual/inter-personal/institutional level to influence WMC skills, 5As) or traditional weight management education
Eichenberg (2023) USA [67]	Mitigating weight bias, incorporating body diversity into clinical care	Multimodal educational session in Health Equity/Social Justice: body diversity (definitions, importance, best practice); addressing weight bias in clinical care, strategies to mitigate personal bias/barriers
Grunvald (2023) USA [68]	Change anti-obesity attitudes	During PC core clerkship: topics commonly encountered in primary care setting; session on obesity biology, pathophysiology, evidence-based therapy recommendations (lifestyle/behavior modification, MI, pharmacotherapy, MBS)
Renold (2023) Switzerland [69]	Reduce weight bias and improve attitudes toward patients with obesity	Elective obesity education course: lecture (environmental/epigenetic factors, psychological approaches, stigma, prevention, ethics, lifestyle change, MBS, pharmacotherapy, childhood obesity); live MBS transmission
Trofymenko (2024) USA [70]	Improve obesity bias among MS	Multi-modality intervention comprising obesity prevalence, strategies to mitigate weight bias, 5As behavior change, MI

*AAMC* Association of American Medical Colleges, *BED* binge-eating disorder, *BIA* bio impedance assay, *BMI* body mass index, *MBS* metabolic and bariatric surgery, *C* control, *E* experimental, *EBM* evidence‐based medicine, *FM* family medicine, *GP* general practitioner, *h* hour, *HP* health professional/s, *MCQ* multiple-choice questions, *MI* motivational interviewing, *min* minute, *mon* months, *MS* medical students, *MME* multi-modal education, *MWM* medical weight management, *PC* primary care, *PH* public health, *PWO* patients with obesity, *Q* questionnaire, *REE* resting energy expenditure, *SP* standardized patient, *WC* waist circumference, *wk* weeks, *WM* weight management, *WMC* weight management counseling, 5*As* (ask, advise, assess, assist, and arrange)

**Table 4:** Goals and content of obesity education interventions in residency programs worldwide

Study	Goal/s	Content
Gonzalez (2006) USA [71]	Develop skills, confidence to counsel PWO/families on healthy eating/PA,	Pediatric obesity prevention program during community health rotation: childhood/adolescent obesity; dietary evaluation and counseling of pediatric patients and parents; nutrition instruction on label content and interpretation; low caloric food and associated cost
Huang (2009) USA [72]	Recognize children at risk for obesity, promote healthy weight	Multidisciplinary education project on prevention, assessment, management of pediatric obesity in outpatient rotation: review expert recommendations/guidelines; epidemiology/etiology, obesity risk, obesity-related conditions; MI/behavioral counseling for dietary/PA change; cultural sensitivity; skills to advocate for weight management; knowledge of practice and delivery systems of weight management
Burton (2010) USA [73]	Improve obesity counseling knowledge, communications skills	Obesity counseling workshop during outpatient ambulatory block: obesity statistics/contributing factors, guidelines, MI principles, 5 As, listening/counseling skills, behavior change
Stahl (2011) USA [74]	Improve behavioral counseling skills for pediatric obesity	Web-based training program, part of community-based education to change children’s eating/PA behaviors: included background information, dietary/PA/screen time recommendations, serving sizes for 2- to 18-year-olds, and strategies for approaching parents and teens using motivational approach and behavioral change planning
Laiteerapong (2011) USA [75]	Improve obesity screening using quality improvement	Quality Assessment and Improvement Curriculum during ambulatory medicine rotation: Rd review charts; identify area for improvement (screen/educate Rd, staff, patients on importance of obesity screening); develop improvements by creating new height, weight, BMI data collection process, patient obesity handout used for preclinic education session, case-based lecture on obesity screening; plan to maintain improvements/obesity education (plan-do-study-act cycles)
Wislo (2013) USA [76]	Improve obesity counseling competence with children/parents	During FM residency on childhood obesity: epidemiology, expert recommendations, using flashcards and games to counsel children/parents on obesity prevention, treatment, PA, nutrition, BMI, in outpatient health centers for low/middle socioeconomic status patients
Jay (2013) USA [77]	Improve obesity counseling skills	Multimodal longitudinal obesity curriculum to train physicians to counsel PWO on 5As model
Acosta (2014) USA [78]	Improve knowledge, attitudes, practice behaviors, clinical outcomes in PWO	Obesity curriculum during noon conference, aligns with guidelines: obesity basic science/diagnosis, epidemiology, pathophysiology, etiology, diagnosis, nutrition/PA evaluation; lifestyle therapy, pharmacotherapy, weight gain prevention, comorbidities, MBS, obesity related psychological disorders, food addiction, behavioral modification; community resources for obesity/stress management
Ren (2016) USA [79]	Increase obesity documentation, counseling	Two-stage intervention during noon conference: Rd provided feedback on extent of documentation/counseling, need for improvement of obesity documentation, importance of weight loss counseling
Iyer (2018) USA [80]	Improve obesity counseling competence in primary care	During ambulatory medicine blocks: obesity epidemiology/PH, causes, prevalence, co-morbidities; dietary assessment/advice; treatment modalities, behavior change, medication, MBS; 5As of obesity counseling, MI theory/techniques; communication/management strategies for challenging clinical scenarios
Carter (2019) USA [81]	Improve obesity detection, counseling, follow-up	Obesity prevention & treatment training curriculum in Rd continuity clinic: calculation of BMI, define overweight/obesity, assess youth PA/eating, screen time; healthy eating/PA strategies, sensitive approach patients/families
Khandalavala (2020) [82] USA	Mitigate obesity bias	Interprofessional obesity teaching modules: obesity etiology, macronutrients, dietary patterns; impact on health care; implicit/explicit bias, Rd self-assess own implicit/explicit bias; obesity management, evidence-based pharmacological/MBS interventions; successful obesity care
Luig (2020) USA [83]	Improve obesity counseling knowledge, confidence	Comprehensive obesity management, part of mandatory Doctor-Patient Relationship course: obesity etiology, 5A’s of obesity management; pregnancy, postpartum; lifestyle changes, medications, MBS; pediatric obesity assessment/management; prevention
Faro (2022) USA [84]	Training for weight management counseling	Communication assessment curriculum for training on weight management counseling; 5As based on obesity management guidelines
Koran-Scholl (2023) USA [85]	Improve obesity bias	Interactive module: 5 clinical vignettes of problematic patient/HP contact in medical home setting, incorporating obesity implicit/explicit bias; obesity causes, biological complexity, obesity bias types, bias recognition, mitigation strategies

*BMI* body mass index, *FM* family medicine, *HP* health professional/s, *MBS* metabolic and bariatric surgery, *MI* motivational interviewing, *PWO* patients with obesity, *PA* physical activity, *Rd* residents, *y* year, 5*As* (ask, advise, assess, assist, and arrange)

Collectively, the OEI employed a wide variety of teaching and learning methods including didactic lectures, web-based self-learning, group discussions, case-based discussions, standardized patient encounters, real patient encounters, personal weight management experience for learners, or wearing of obesity-simulation empathy suit [11, 27–85] (Supplementary Tables 2 and 3). In terms of timing of the OEI, for medical students, about half were during the first, second, or third year of medical school, while roughly a third were over multiple years. In residency programs, OEI were mostly delivered within family medicine, internal medicine, or pediatric programs.

### Obesity-Related Domains and Identified Gaps of the Educational Interventions

Table 5 shows the range of obesity-related domains addressed by educational interventions. Section (A) of the table shows that for medical students, the most common domains comprised the basic sciences of obesity (48.3%), counseling, and nutrition (45% for each). Section (B) depicts that for residents, the most common content addressed was basic sciences of obesity and counseling (21.6% for each).

**Table 5:** Obesity-related domains addressed by educational interventions (*N* = 60)

Study	EHPP	BS of obesity assessment & diagnosis	Nutrition	PA	Behavioral	Counseling ^*a*^	Pharma	MBS	Weight Stigma ^*b*^	Ethics/Professionalism	Health Literacy	Practice Guidelines
**(A) Medical schools**												
Dengerink (1982) [27]					**✔**							
Wiese (1992) [28]									**✔**			
Rourke (1999) [29]		**✔**										
Hodgson (2000) [30]		**✔**	**✔**		**✔**							
Banasiak (2001) [31]		**✔**	**✔**	**✔**			**✔**	**✔**				
Buchowski (2002) [32]			**✔**	**✔**								
Carson (2002) [33]		**✔**	**✔**									**✔**
Carson (2003) [34]			**✔**	**✔**		**✔**						
Conroy (2004) [35]			**✔**	**✔**		**✔**						
Poirier (2004) [36]					**✔**	**✔**						
Endevelt (2006) [37]	**✔**	**✔**	**✔**	**✔**			**✔**	**✔**				**✔**
Rodríguez (2006) [38]			**✔**	**✔**	**✔**	**✔**						
Barss (2008)[39]			**✔**	**✔**								
Bell (2008) [40]						**✔**						
Dayal (2008) [41]		**✔**	**✔**									
Zoberi (2008) [42]	**✔**	**✔**	**✔**	**✔**	**✔**		**✔**	**✔**				
Moser (2009) [43]	**✔**				**✔**	**✔**						
Schroder (2010) [44]					**✔**							
Roberts (2011) [45]		**✔**						**✔**				
McAndrew (2012) [46]		**✔**	**✔**	**✔**	**✔**	**✔**						
Miller (2012) [47]	**✔**	**✔**	**✔**	**✔**	**✔**	**✔**	**✔**	**✔**	**✔**			**✔**
Poustchi (2013) [48]									**✔**			
Schmidt (2013) [49]		**✔**	**✔**	**✔**	**✔**							**✔**
Birkhead (2014) [50]			**✔**		**✔**	**✔**						
Kushner (2014) [51]						**✔**			**✔**			
Matharu (2014) [52]									**✔**			
Brown (2015) [10]		**✔**	**✔**	**✔**	**✔**	**✔**						**✔**
Lee (2015) [53]		**✔**										
Milford (2016) [54]	**✔**	**✔**	**✔**	**✔**		**✔**					**✔**	
Chisholm (2016) [55]		**✔**			**✔**	**✔**						
Pasarica (2016) [56]		**✔**	**✔**		**✔**	**✔**						**✔**
Wilechansky (2016) [57]	**✔**	**✔**	**✔**	**✔**		**✔**						
Gayer (2017) [58]	**✔**	**✔**	**✔**	**✔**	**✔**	**✔**	**✔**	**✔**				**✔**
Hawa (2017) [59]		**✔**			**✔**	**✔**						
Pasarica (2017) [60]	**✔**	**✔**	**✔**	**✔**	**✔**	**✔**	**✔**	**✔**				**✔**
Ryan (2017) [61]		**✔**	**✔**	**✔**	**✔**	**✔**			**✔**			
Broad (2018) [62]	**✔**	**✔**	**✔**	**✔**		**✔**						
Geller (2018) [63]		**✔**							**✔**	**✔**		
Cohen (2019) [64]		**✔**				**✔**			**✔**			
Leedham-Green (2019) [65]		**✔**	**✔**		**✔**	**✔**						**✔**
Ockene (2021) [66]		**✔**	**✔**		**✔**	**✔**						
Eichenberg (2023) [67]	**✔**	**✔**			**✔**	**✔**			**✔**			
Grunvald (2023) [68]		**✔**	**✔**	**✔**	**✔**	**✔**	**✔**	**✔**				
Renold (2023) [69]	**✔**	**✔**	**✔**	**✔**	**✔**	**✔**	**✔**	**✔**	**✔**	**✔**		
Trofymenko (2024) [70]	** ✔**					** ✔**			**✔**			
**Total, *n* (%)**	**12 (20)**	**29 (48.3)**	**27 (45)**	**20 (33.4)**	**23 (38.3)**	**27 (45)**	**8 (13.3)**	**9 (15)**	**11 (18.3)**	**2 (3.3)**	**1 (1.6)**	**9 (15)**
**(B) Residency programs**												
Gonzalez (2006) [71]	**✔**	**✔**	**✔**			**✔**						
Huang (2009) [72]	**✔**	**✔**	**✔**	**✔**	**✔**	**✔**			**✔**	**✔**		**✔**
Burton (2010) [73]	**✔**	**✔**			**✔**	**✔**						**✔**
Laiteerapong (2011) [75]		**✔**										
Stahl (2011) [74]	**✔**	**✔**	**✔**	**✔**	**✔**	**✔**					**✔**	
Jay (2013) [77]						**✔**						
Wislo (2013) [76]	**✔**	**✔**	**✔**	**✔**		**✔**					**✔**	**✔**
Acosta (2014) [78]	**✔**	**✔**	**✔**	**✔**	**✔**	**✔**	**✔**	**✔**				**✔**
Ren (2016) [79]		**✔**				**✔**						
Iyer (2018) [80]	**✔**	**✔**	**✔**	**✔**	**✔**	**✔**	**✔**	**✔**				
Carter (2019) [81]	**✔**	**✔**	**✔**	**✔**		**✔**					**✔**	**✔**
Khandalavala (2020) [82]		**✔**	**✔**	**✔**	**✔**	**✔**	**✔**	**✔**	**✔**			
Luig (2020) [83]	**✔**	**✔**	**✔**	**✔**		**✔**	**✔**	**✔**				
Faro (2022) [84]						**✔**						**✔**
Koran-Scholl (2023) [85]		**✔**							**✔**			
**Total, n (%)**	**9 (15)**	**13 (21.6)**	**9 (15)**	**8 (13.3)**	**6 (10)**	**13 (21.6)**	**4 (6.6)**	**4 (6.6)**	**3 (5)**	**1 (1.6)**	**3 (5)**	**6 (10)**

Due to space limitations, only the first author is cited

^*a*^includes motivational interviewing

^*b*^includes weight stigma, bias, and prejudice

*BS b*asic sciences, *EHPP* epidemiology, health policy, prevention, *PA* physical activity, *Pharma* pharmacotherapy, *MBS* metabolic and bariatric surgery

**Fig. 4:**
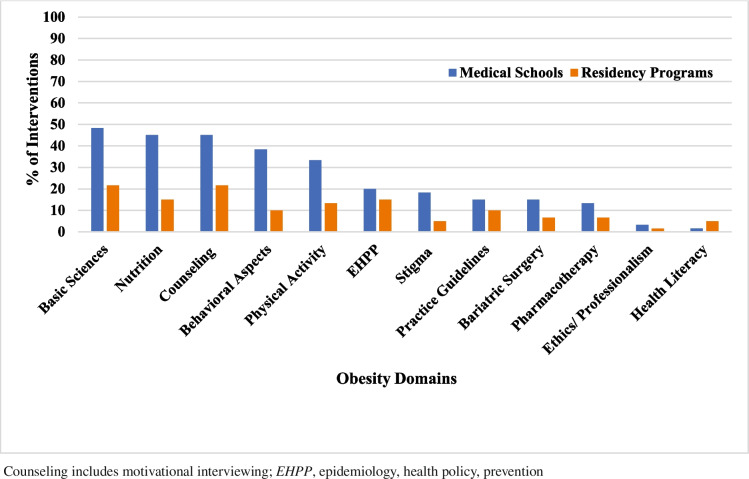
Percentage of obesity-related domains covered in the 60 educational interventions

Generally, there was limited coverage of all the obesity-related domains for both medical students and residents, with the coverage being much less across residency programs compared to medical schools. In medical schools, the domains least covered were pharmacotherapy (13.3%), ethics/professionalism (3.3%), and health literacy (1.6%). In residency programs, there was low coverage across most domains, with the least common domains covered being MBS and pharmacotherapy (6.6% each) weight stigma and health literacy (5% for each), and ethics and professionalism (1.6%). Figure 4 illustrates the obesity-related domains covered in the 60 educational interventions.

**Fig. 5:**
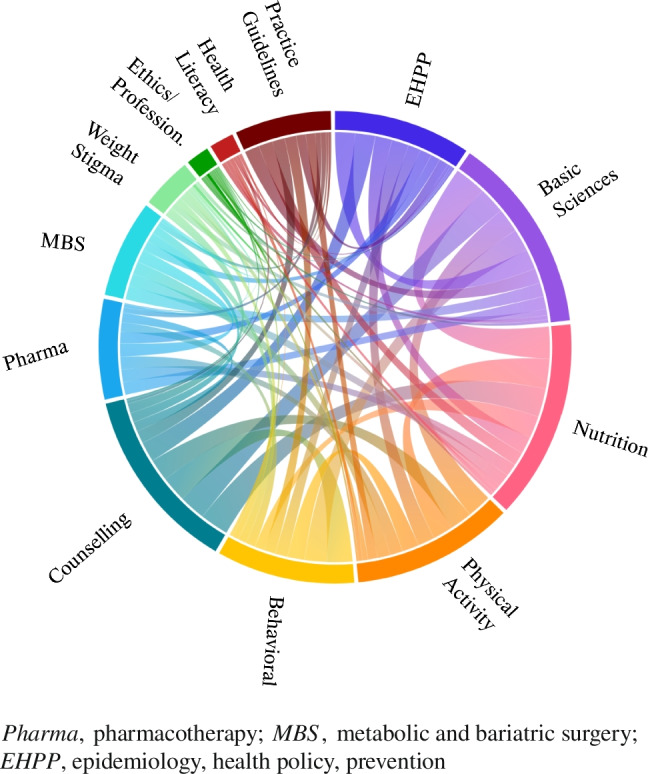
Visualization analysis of the domains of obesity across the identified interventions

The chord diagram visualizes the relationships between the different obesity-related domains across the identified OEIs by displaying the links between domains (Fig. 5). Domains are represented as segments around the circular edge, and a segment’s length indicates the extent to which a given domain is represented in the published literature. The thickness of the chords (curved lines connecting segments) shows the strength of the connections, highlighting the likelihood that given domains would be present together in an OEI. For instance, the two domains “Basic Sciences” and “Nutrition” have a strong connection with each other, indicated by a prominent chord; “Behavioral” is linked strongly with “Counseling” and “Basic Sciences”; “Nutrition” interrelates with “Physical Activity”; “Epidemiology/Public Health/Health Policy” (EHPP) is broadly linked to many domains including “Counseling” and “Basic Sciences.” Conversely, the diagram shows relatively weak connections for certain domains, e.g., “Bariatric Surgery” (MBS), “Health Literacy,” and “Ethics/Professionalism.”

### Alignment of Identified Domains with Guiding Framework

**Fig. 6:**
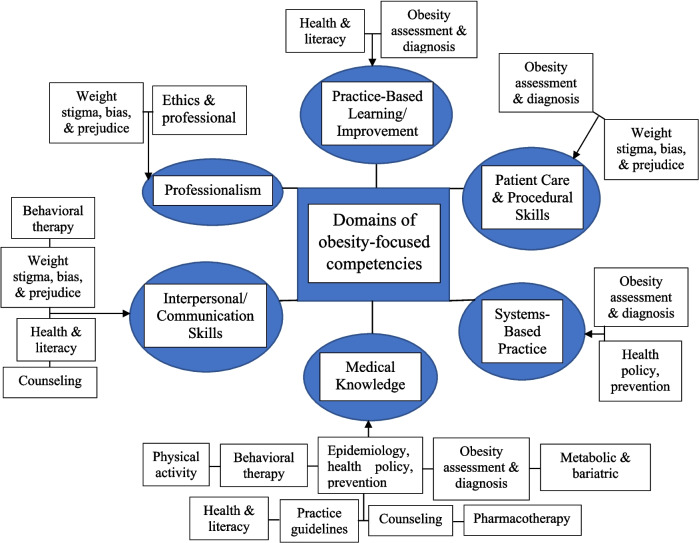
Obesity-related domains identified by the review and their alignment with the guiding framework (adopted from [12, 18])

Figure 6 shows the obesity-related domains we identified from the content of the OEIs and their alignment with the OMEC and ACGME-I framework and competencies employed in the current study. Some obesity-related domains identified aligned with multiple competencies from the OMEC and ACGME-I frameworks.

## Discussion

More than a decade ago, the Association of American Medical Colleges concluded that medical education must assure that future physicians are better prepared to provide effective care for PWO, and hence obesity prevention and treatment should be included in medical school curricula [86]. Since then, with the rising prevalence of obesity and its recognition as a disease [87], some progress has been achieved in incorporating obesity education into undergraduate medical education and graduate medical education curricula [17, 86]. However, despite this, medical students and residents remain inadequately prepared to manage obesity [88].

To date, no study has undertaken and explored the content of published OEI delivered to young physicians to evaluate whether such content meets the educational needs required to provide the knowledge, skills, and attitudes necessary for optimal care of PWO. The present study undertook this task. Our main findings were that most of the 60 published OEI we identified were implemented in North America. Most OEI were dedicated to medical students, much less were available for residents, and none was delivered to fellows. As for content, there was limited coverage of the 12 obesity domains for both medical students and residents; however, within the limited coverage, medical schools had broader coverage of the domains compared to residency programs. The most common obesity domains taught to medical students included the basic sciences of obesity (48.3%), counseling (45%), and nutrition (45%), while for residents, the most common content addressed were basic sciences of obesity (21.6%) and counseling (21.6%). Wide variations were observed in the structure and delivery of the obesity curricula. Below we discuss these points in detail, addressing some of the gaps noted across the OEI that were identified.

In terms of the numbers of published OEI delivered to medical students and residents, we observed a considerable increase (*n* = 60), where earlier reviews reported a range of 12–31 interventions [14–16]. Such an increase is commensurate with the surge in the obesity epidemic and its recognition as a chronic disease. However, geographically, this increase in published OEI appeared to be primarily in North America.

Pertaining to content, the current review uncovered several important gaps. For instance, the meager focus on pharmacotherapy in the OEI delivered in medical schools (13.3%) and residency programs (6.6%) agrees with a recent survey of obesity curricula in medical schools, where 31% of the surveyed schools reported very little or no coverage of pharmacotherapy in their curricula [89]. This is despite that 78% of internal medicine program directors acknowledge that pharmacotherapy is “very” or “fairly” important [11]. Pharmacotherapy is a core obesity topic and a safe and effective obesity treatment modality [90]. With the approval of many new anti-obesity medications [91, 92], future physicians require solid education on their indications, effectiveness, dosage, side effects, and safety [93].

Likewise, the present review found that MBS received sparse attention, being included in only 15% of OEI in medical schools and 6.6% of residency programs. Our findings support the literature. In the USA, a survey found that MBS was covered to a “great extent’ in the curriculum in only 22% of residency programs [11]. Similarly, in Poland, a survey of 468 final-year students at four medical universities revealed that students had limited MBS knowledge, where only 4 students achieved the maximum number of correct answers, and the majority had not received sufficient MBS education, expressing a need to expand the curriculum to include more information on MBS [94]. In Saudi Arabia, while 82% of medical students had good MBS theoretical knowledge, only 17.4% of interns did so, reporting a need to improve their knowledge and expand the curriculum [95]. In agreement, final-year medical students in Turkey who received a distinct MBS educational program answered questions regarding surgical indications, efficacy, and patient referral more accurately than those who did not, suggesting that incorporating more MBS knowledge will impact future patient care [96]. MBS is a very effective and widely utilized obesity treatment modality and a core obesity competency topic warranting more attention in OEI than currently is, as graduating physicians will surely attend to PWO during their practice, seemingly lacking the necessary skills.

In terms of weight bias/stigma, the current study observed that OEI addressing weight stigma were limited in both medical schools (18%) and residency programs (5%). These findings support a study where less than one-tenth (8.6%) of residency programs covered weight stigma [11], despite that explicit and implicit weight bias were commonly reported among medical students and residents [97–99]. Weight bias in health care negatively affects PWO, resulting in low levels of physician–patient rapport, avoidance or delay of health services, and lack of trust in, as well as low satisfaction with care providers [98, 100]. Moreover, patients who experience weight bias are more likely to have maladaptive eating behaviors, low physical activity levels, and higher anxiety and depression [98, 101, 102]. Addressing such weight stigma is a core obesity competency topic, and education represents a key approach to minimize stigma and its impacts [100].

As for ethics and professionalism, we again observed very low coverage of this domain across the OEI in both medical schools (1.3%) and residency programs (1.6%), in agreement with other research [11]. Such deficiency has adverse effects on clinical practice and patient outcomes [63, 97]. Commitment to professionalism and adherence to ethical principles are core competencies in obesity education, as physicians should demonstrate ethical behavior and integrity, and display compassion and respect when counseling PWO, as well as their families.

Regarding health literacy, the present review noted insufficient coverage of this domain across the identified OEI, amounting to a scarce 3.3% of OEI in medical schools and 1.6% in residency programs. Health literacy is critical for effective health communication, with major implications for individuals’ health. It is also a crucial element in training future physicians as poor health literacy is a determinant of poor health outcomes and higher costs [103–105]. The Institute of Medicine recommends teaching health literacy to medical students, although standards are not clearly established for implementing such training into medical curricula [106].

### Integrating Obesity Content in Medical Education: Challenges

Integrating comprehensive obesity education that addresses the wide range of obesity-related topics in medical schools and residency programs faces numerous challenges [11–13]. These include overcrowded curricula, time constraints within already packed curricula, lack of faculty expertise and poor faculty knowledge, and to a lesser extent, lack of student interest, as well as overall negative attitudes of physicians pertaining to the disease of obesity [89]. Priority is a further challenge, as among 141 U.S. medical school deans, an evaluation of the current status of obesity education showed that half of medical school deans felt that obesity education was a low priority or not a priority [89]. Such findings highlight the limited coverage of obesity education and the lack of prioritization to develop expanded curricula in obesity [89]. Thus, novel interventions are required to adopt interprofessional education models, incorporating state-of-the-art learning technologies and patient-centered narratives. The aim is to engage medical students alongside nutritionists, psychologists, and exercise physiologists, for holistic understandings of obesity management, with the use of technologies, virtual simulations, and a patient-centered focus to enhance empathy and clinical competencies. Moreover, robust institutional commitment to curricular reforms is critical in equipping future health care providers with the skills needed to combat the obesity epidemic effectively and equitably.

### Alignment of Identified Domains with Guiding Framework

Most OEI domains we identified aligned directly with the OMEC and ACGME-I guiding framework. Figure 6 illustrates that several domains we identified, “Epidemiology, Health Policy and Prevention,” “Basic Sciences of Obesity Assessment & Diagnosis,” “Nutrition,” “Physical Activity,” “Behavioral,” “Pharmacotherapy,” “Bariatric Surgery,” and “Practice Guidelines” all aligned with framework’s domain “Medical Knowledge” and its competencies. Likewise, we noted that student-designed material and activities for patients and families, e.g., educational modules, sessions, and workshops using presentations and games [10, 38, 46, 54, 62, 75, 76] all provided evidence of engagement of medical students and residents in teaching and education, aligning with framework’s domain “Practice-Based Learning and Improvement” (effectively educating patients on the disease of obesity). Our observations of OEIs where students paired with patients undergoing MBS and established longitudinal relationships [45], or where students attended meetings on overeating and reported their experiences of the meetings [44], aligned with the framework’s domain “Professionalism” (displays compassion and respect toward all patients and families who are living with overweight or obesity).

### A Way Forward

Recently, many educational initiatives have been implemented to bridge the challenges in obesity education within medical schools and residency programs and to develop comprehensive obesity education across the continuum of undergraduate and graduate medical education. For example, the Provider Training and Education Workgroup at the National Academies developed ten high-level obesity prevention and management competencies for health care professional schools [107]. In addition, The Obesity Medicine Education Collaborative (OMEC) initiative by the Obesity Medicine Association, Obesity Society, and American Society of Metabolic and Bariatric Surgery developed 32 measurable obesity-related competencies and associated benchmarks across the six core domains of ACGME [12]. Likewise, the Obesity Canada Education Action Team, based on the CanMEDS framework, created the Canadian Obesity Education Competencies comprising seven professional roles, 13 obesity-focused key competencies, and 37 enabling competencies [108]. Similarly, for fellowship training, the Accreditation Council for Graduate Medical Education—international (ACGME-I) developed advanced specialty program requirements for graduate medical education in obesity medicine/bariatric medicine for family medicine, internal medicine, and pediatrics [18]. Collectively, such initiatives could provide appropriate guidance and structure for obesity education curricula.

Based on the knowledge gaps that the current review uncovered, future strategies would benefit from a multipronged approach. In terms of published research, across the four decades that our searches covered, we identified only 60 OEI studies published since 1982. Hence, we encourage educators and researchers worldwide to publish their experiences and interventions pertaining to furthering and refining obesity education. Particularly deficient were OEI being implemented outside of North America, as these were unrepresented in the identified studies.

In terms of target learners, despite the general OEI insufficiency we observed, more of those were dedicated to medical students, and much less were available for residents. Thus, medical schools’ deans, program directors, faculty members, and educators need to prioritize obesity education for medical students, even more for residency programs, and with immediate and urgent attention to fellowship curricula as the present review identified no published OEI delivered to fellows, an advanced level of physicians that need to be highly prepared to care for PWO.

As for the content of OEI, it needs to integrate and incorporate the many interlacing domains of obesity knowledge, competencies, and skills across the existing curricula. Such multidisciplinary curricula should incorporate obesity-related basic sciences, clinical sciences, as well as public health, policy, and preventive aspects. Premised on the extant literature, Table 6 proposes a range of components required for an all-rounded obesity curriculum as potential guidance to enhance and further the education and training of medical students, residents, and fellows.

**Table 6:** Obesity-related domains for future educational interventions

Domain^*a*^	Subdomains and brief description
Epidemiology, health policy, prevention	**-** Epidemiology, public health policies and initiatives pertaining to obesity **-** Social, community, and environmental change to reduce obesity epidemic **-** Primary, secondary, and tertiary obesity prevention in adults, children, pregnancy and postpartum **-** Health care discrimination; health equity
Basic sciences of obesity assessment	**-** Definition, classification and staging: overweight and obesity **-** Physiology: energy homeostasis, weight regulation **-** Etiology and pathogenesis of obesity **-** Obesity related comorbidities, benefits of BMI reduction **-** Bariatric-focused history, physical examination, diagnostic testing, clinical assessment of energy expenditure
Medical weight management	Multidisciplinary care: Includes weight gain prevention strategies; management of comorbidities; referral and collaboration with other HCP; emerging obesity treatment modalities; maintaining lifestyle change
Nutrition/diet	**-** Nutrition: micro/macronutrients, daily energy requirements, dietary reference intake standards **-** Importance of nutrition for various body-organ systems; impact of nutrition on health and disease **-** Dietary assessment - Nutrition interventions to develop a comprehensive, personalized obesity management care plan
Physical activity	**-** Basic skeletal muscle anatomy and physiology; Difference between physical activity, exercise, and non-exercise activity - Types of training and associated benefits - Risk of exercise, recommendations for stress testing - Behavior change, barriers to exercise - PA interventions to develop a comprehensive, personalized obesity management care plan, exercise prescription
Behavioral aspects	**-** Theories of behavior, stages of behavior change **-** Behavioral modification interventions, e.g., cognitive behavioral therapy and acceptance and commitment therapy **-** Psychological disorders related to obesity **-** Assessment, screening and management for eating disorders, mood and sleep disorders
Motivational interviewing/counseling	**-** Barriers to effective consultations **-** Communication skills, 5As counseling approach (ask, advise, assess, assist, arrange) **-** Counseling interventions and motivational interviewing for behavior and lifestyle changes
Pharmacotherapy	**-** Pharmacological treatments of obesity as part of comprehensive, personalized obesity management care plan **-** Anti-obesity medications; indications, effectiveness, mechanism of action, dosage, side effects, safety, monitoring - Weight promoting medications and their alternative medications
Bariatric surgery and endoscopic procedures	**-** Types, effectiveness, indications, risks and benefits **-** Preoperative evaluation: nutritional, psychological and medical assessment **-** Post bariatric medical/surgical follow up; addressing weight regain **-** Endoscopic procedures: indications, contraindications, risk and benefits
Stigma/weight bias	**-** Definitions, implicit and explicit obesity bias, impact on health care access and clinical care **-** Body diversity/acceptance, their relevance to clinical care **-** Mitigation strategies: approaches to reduce weight bias/stigma in health care settings **-** Non-judgmental communication and language usage with PWO
Ethics/professionalism	**-** Ethics and professionalism in care of PWO **-** Addressing disrespectful behavior towards patients with obesity
Healthy literacy	**-** Importance of HL for patients and physicians; HL in patient/provider communication **-** Lower HL as barrier to behavior change **-** HL strategies
Practice guidelines	**-** Obesity management guidelines **-** Dietary guidelines **-** PA guidelines **-** MBS guidelines

^*a*^Guided by [12, 18]

*BMI* body mass index, *HL* healthy literacy, *HCP* health care professionals, *PA* physical activity, *PWO* patients with obesity, *MBS* metabolic and bariatric surgery

This study has limitations. The studies identified in the current review do not necessarily represent all available OEIs worldwide. We are unable to exclude the possibility of other OEIs implemented at various institutions but not published. Hence, any generalizability of the findings needs to exercise caution. This is a limitation common to any review, and we strongly encourage medical schools, institutions, and residency programs to publish their OEIs to enhance the comprehensiveness of future reviews. Despite these limitations, the study has many strengths. To our knowledge, this is the first review to scope OEIs for medical students and residents globally. It reviewed a substantial number of published OEIs spanning over four decades, and meticulously assessed their details, evaluating the educational goals, content, and the obesity-related domains addressed. It also mapped the specific components of each OEI to recently published international educational and competencies frameworks, allowing the identification of trends and gaps. Moreover, the review examined the intended target populations, teaching/learning methods, health professionals involved in teaching, duration of the interventions, and evaluation tools used. The review highlighted potential actionable strategies to enhance the scope and comprehensiveness of obesity education worldwide.

## Conclusion

As obesity rates continue to rise worldwide, addressing this public health challenge is a critical priority. Despite this, no published obesity educational interventions target fellows, and the curricular content of obesity education initiatives delivered to medical students and residents appears to be inadequate. There is generally limited coverage of most obesity domains across educational interventions for medical students, and even more so in those specifically tailored for residents. Insufficient curricular content was particularly noted in relation to pharmacotherapy, metabolic and bariatric surgery, stigma, ethics and professionalism, as well as health literacy. Multipronged future strategies should encompass prioritizing obesity education and integrating multiple knowledge, competencies, and skills domains across the curricula. Collectively, such actions could contribute to a future generation of physicians better equipped to care for PWO.

## Supplementary Information

Below is the link to the electronic supplementary material.

Supplementary file1 (DOCX 83 KB)

## Data Availability

No datasets were generated or analyzed during the current study.
