# Supplementary material for: The Landscape of Obesity Education Worldwide — Are We Doing Enough? Scoping Review of Content of Obesity Educational Interventions in Medical Schools and Residency Programs
Source: Obes Surg. 2025 Mar 3;35(4):1201–22. doi: 10.1007/s11695-024-07654-y (PMC11976346; doi:10.1007/s11695-024-07654-y)
Supplement: Supplementary file 1 — Supplementary file1 (DOCX 83 KB) [file 11695_2024_7654_MOESM1_ESM.docx]

**Supplementary Table 1.** Terms Used Within Systematic Review Search Strategy

| 1. Obesity 2. Overweight 3. Education 4. Medical Schools 5. Medical Student 6. Residents 7. Residency Program 8. Fellow 9. Fellowships Program 10. Curriculum 11. Trial 12. Knowledge 13. Confidence 14. Attitude 15. Skills |
| --- |

**Supplementary Table 2** Additional characteristics of obesity education interventions in medical schools worldwide

| **Study** | **Teaching/ Learning methods, HP involved** | **Duration/ TA** | **Evaluation tool/s** |
| --- | --- | --- | --- |
| Dengerink (1982)  USA [27] | Lecture; review article on obesity treatment; MS assigned to patient with obesity to assist in behavior change  HP involved: Psychology faculty | 2 h lecture; patient meeting for 8-9 wk; N=20, 1^st^ y MS | Post intervention patient questionnaire; Post intervention MS questionnaire |
| Wiese (1992) USA [28] | Video; audio; written components; role- playing exercises.  HP involved: NR | Duration: NR  N**=**75, 1^st^ y MS | Questionnaire for MS before/ after workshop, and 1 year later |
| Rourke (1999)  USA [29] | Educational sessions (face to face); Total body composition assessment; Presentation by MS to class  HP involved: NR | 1 h  N=69, 2^nd^ y MS | Post intervention test; Post intervention measurement of anthropometric and body composition of MS |
| Hodgson (2000)  USA [30] | Lectures; lab exercises; dietary self-assessment; small-group discussions; SP interviews, PBL  HP involved: nutrition education members | Curriculum over 4 y  N=148, 1^st^, 3^rd^, 4^th^ y MS | Pre/post-test survey; 4 delayed posttests at 1^st^ to 4^th^ y |
| Banasiak (2001)  USA [31] | Clinical clerkships rotations  HP involved: NR | Duration: NR  N=201, 2^nd^/ 3^rd^ y MS | Pre/post questionnaire |
| Buchowski (2002)  USA [32] | Computer-based interactive curriculum; animations, quizzes, practice cases; consultation; simulated patient case videos  HP involved: physician, dietician, instructor, virtual physician mentor | 12 wk  N=80, 1^st^ y MS | Pre/post MCQ; end of course questionnaire |
| Carson (2002)  USA [33] | Mandatory lectures twice/wk; weekly clinic participation; resource materials, web-based cases; class discussion; computer-simulated patients; MCQs with feedback; submission of assessment/ plan of patients.  HP involved: physician, dietician | 99.3 h, 4 wk rotation  N=196 4^th^ y MS, (C=40, 37% F; E=156, 42% F) | Pre/post questionnaire; audit of primary care clinic chart notes |
| Carson (2003)  USA [34] | Distribution of color- coded tape measure; pocket reference card; computer-based cases; interactive questions.  HP involved: NR | 4 wk, 10, 4^th^ y MS | Pre/post MCQ; MS self-report whether they measured WC |
| Conroy (2004)  USA [35] | Lectures; small-group PBL tutorials; clinical cases; simulated cases for counseling skills; student debates; MS assessed own health behaviors with survey and personalized feedback about their results; completed and analyzed 24-hour diet record and reviewed it with a dietitian  HP involved: dietician | 8 h, 90 min tutorial  N=137, 2^nd^ y MS | Pre-/post assessment; follow-up survey |
| Poirier (2004)  USA [36] | Didactic presentation, group exercises; reflective listening using vignettes; reading material; counseling intervention checklist for behavior change with feedback about interviewing skills; instructional video featuring professional counselors roleplaying confrontational counseling with interactive discussion with faculty; group role plays; practice counseling skills with simulated patient  HP involved: internist, psychiatrist/ psychologist, counselors, faculty | 10 h  N=42, 1^st^ y MS | Pre/post questionnaire; MCQ |
| Endevelt (2006)  Israel [37] | Reading assigned articles; Lecture; interactive case-discussions; problem-solving session; integrative nutrition workshop; Further research on a treatment modality as homework with class discussions  HP involved: NR | 10 h workshop  N=122, 2^nd^ y MS | MCQ, questionnaire to assess program |
| Rodríguez (2006)  USA [38] | Lectures & didactics; outpatient clinical experience; case-based teaching; MS collect data, interpret results, present outcomes in oral presentation for peers/ faculty  HP involved: NR | 8-12 h/ wk over 4 wk  N=18, 3^rd^/4^th^ y MS | MS evaluation of project; patients’ questionnaire |
| Barss (2008)  UAE [39] | 5 lectures; MS conduct home interview (family’s lifestyle history, lifestyle behaviors), formulate recommendations; MS assess their own lifestyle (1-week PA log, nutritional intake, BMI); literature searching; oral presentations, MS chooses topic to research, present to peers/faculty) with questions/ comments.  HP involved: community medicine faculty, medical education | 2 mon duration  N=130, 1^st^ y MS | MS: questionnaire; personal log; oral presentation |
| Bell (2008)  USA [40] | Short didactics; video demonstrations; group role plays; interactive exercises with reflection; video of physician interviewing patient using MI, with group discussion; role play vignettes with “resistant” patients; instructor feedback; students present their patient interviews videos, with peer review.  HP involved: physician, social worker, graduate student educator | 8 h in 4-wk curriculum  N=53, 3^rd^ y | Pre/post assessments; post MS commitment statements, survey 3 mon later to check if commitments implemented |
| Dayal (2008)  USA [41] | Case-based module; workshop in ob/gyn clerkship; PowerPoint presentation; group discussion: case presentation  HP involved: senior faculty | 2 h workshop  N=113 -151, 3^rd^ y MS | Pre/post survey (during 4^th^ y medical school) |
| Zoberi (2008)  USA [42] | Didactic lecture  HP involved: family physician, psychologist | 90 min  N=84, 3^rd^ y MS | Pre/ post test |
| Moser (2009) USA [43] | Classroom instruction and clinical/ personal experiences: MI/patient education workshops; a teaching observed standardized clinical examination; stages of change video, behavior change exercise; reflective journals; interactive lectures; team-based learning; patient case presentations; role-play; real/ SP interview; student-as-patient exercise; self-care assessment; individual wellness plans; community project; ambulatory experiences  HP involved: IM/FM faculty, psychiatry, pediatrics | 60-h over 4-wk  N=50, 3^rd^ y MS | End-of-rotations surveys to evaluate module |
| Schroder (2010)  New Zealand [44] | MS attended and observed overeaters meeting in the community, submit reflective report  HP involved: overeaters group representatives | Duration: NR  N=72 5^th^ y MS | MS reports on experiences of attending overeaters meetings, thematic analysis |
| Roberts (2011)  USA [45] | MS paired PWO undergoing MBS establish longitudinal relationship; online curriculum; interdisciplinary patient visits; clinical skill building; faculty mentorship (discuss patients, present relevant papers, ask questions, interact with faculty about their clinical/ research interests); reflections journal. HP involved: clinical/ research faculty | 1 y  N=13, 3^rd^ y MS, 4 MS were intervention group; 9 C not enrolled in pilot program | Pre/ post survey (third y); qualitative reflection essay, thematic analysis |
| McAndrew (2012)  USA [46] | MS-created educational module; interactive didactic presentation; group discussion; Team role play.  HP involved: pediatric endocrinologist | 1 h  N=25 1^st^, 2^nd^ y MS, over 2 y | Open-ended surveys: teachers, MS, HS students |
| Miller (2012)  USA [47] | Web-based teaching and learning modules: PowerPoint presentations; include text, audio narration, figures, graphics, animations, video; Pre/post-quiz  HP involved: web-based | 10–15 min for each of 8 modules  N=NR, 1^st^, 2^nd^/3^rd^/4^th^ y MS | Post intervention evaluation of modules |
| Poustchi (2013)  USA [48] | Video presentations; Interactive discussion.  HP involved: obesity experts | 1 h  N=64, 2^nd^/3^rd^ MS, 68.2% F | Pre/post surveys |
| Schmidt (2013)  USA [49] | MS participate in week­ly FM clinic weight management class; complete personal weight management experience; After 4 wk, MS reassess their weight, ef­fectiveness of their plan.  HP involved: registered dietitian | 4 wk  N=75, 3^rd^ y FM clerkship, 51% female | MS reflections, thematically coded; MS reported own objective measurements e.g., weight, BMI, WC |
| Birkhead (2014)  USA [50] | MS directed culinary classes: presentation, ran cooking lesson, guided dinner discussion, participants and MS discussed new techniques/ information they learned and how to incorporate them into their daily lives.  HP involved: physician, trained chef | 2 h  N=125, 1^st^/2^nd^ y MS | Post intervention MS survey |
| Kushner (2014)  USA [51] | Reading/ reviewing articles with faculty preceptor; SP clinical encounter; short patient scenarios for role play; SP provided feedback on verbal/ non-verbal communication skills; peer feedback on performance; faculty facilitated discussion of SP interaction; small group discussion.  HP involved: faculty preceptor | About 1 h  N=127, 1^st^ year MS, 47% female | MS questionnaire completed before, after and 1 y; MS Reflections |
| Matharu (2014)  USA [52] | Educational intervention: students assigned to standard lecture (PowerPoint presentation) with opportunities to ask questions or play reading  HP: faculty | 1 hour  N=129 | Pre/post surveys |
| Brown (2015)  USA [10] | Educational/ clinical: didactic lessons; online videos; students deliver 10-wk weight loss class to community using manual developed with multidisciplinary WMC advisors; group meetings, interactive discussions/ feedback from advisors  HP involved: WMC advisors | 20 wk  N=35, medicine, nursing, graduate studies, HP students | Post intervention data on weight loss outcomes, compared to professional-led obesity program |
| Lee (2015)  Canada [53] | Game-based learning using board/ other games; interactive power point; MCQ; activity with discussion for deeper learning; question bank to motivate learning about impact of obesity  HP involved: faculty members, residents, graduate students | 90 min  N=42, 2^nd^ y MS | Pre/post online surveys |
| Milford (2016)  USA [54] | Weekly didactic sessions before intervention; MS as educators/ mentors for children, parents, staff: measure child’s pre-intervention BMI, participate in activities, attend parent trainings, assess health literacy; explain BMI numbers using carefully chosen language; lead nutrition/PA goal setting; engage with families through Facebook with health-literate nutrition/ PA tips, recipes  HP involved: dietician, pediatrician | 5 mon  12, 1^st^/2^nd^ y MS | Pre/post survey (7 mon); open-ended journal, thematic analyses; parents/ staff complete survey |
| Chisholm (2016)  UK [55] | Obesity management session; group discussion; video of real patient stories; practice skills in large group with video patient scenario; role-play with peer/ tutor feedback; reflection on new skills  HP involved: GPs as tutors | 3 h session, N=41, final 2 y MS, 61.8% F | Phone/email feedback of MS/ tutors, thematic analysis; open questions to participants |
| Pasarica (2016)  USA [56] | Active EBM session, class discussion facilitated by MCQ; group work, MS solve scenario of patient wanting to use commercial weight-loss program; MS access Internet, appraise EBM articles, present findings, answer questions, receive formative feedback  HP involved: instructor with clinical nutrition expertise | 1 h, N=112, 2^nd^ y MS | Post survey, two open-response questions to assess module |
| Wilechansky (2016)  USA [57] | Web-based interactive learning module: decision making scenarios, lectures, animated characters to simulate human interactions, MCQ; medical case scenario; narration, reading, diagrams, case scenarios, quizzes; Hyperlinks to web-based information  HP involved: NR/ web base module | 60-90 min, N=64, 3^rd^ y MS | Post survey |
| Gayer (2017)  USA [58] | Lectures; online interactive case study simulations; MCQ; virtual patient case presentations  HP involved: NR | 1^st^ y, 5 h; 2^nd^ y, 3 h  N= 718 | Pre/post survey; post curriculum MCQ |
| Hawa (2017)  Canada [59] | Module as individual study or small-group exercise: video clips of evolution of cases; clinical application exercise; Interactive quizzes, video clips; evidence-based literature; reflective questions  HP involved: NR | 10-12 wk  N=10 3^rd^ y MS | Written/ verbal feedback from faculty and students |
| Pasarica (2017)  USA [60] | Web base self-learning module; individual or collaborative exercise during mandatory didactic session  HP involved: self-learning module | 20-30 min over 15 mon  N=180, 3^rd^/4^th^ MS | Post survey on module effectiveness |
| Ryan (2017)  USA [61] | PowerPoint presentations  HP involved: NR | 1 h seminar,  N=24 under/graduate health students, 62.5% F | Pre/post MS questionnaire |
| Broad (2018)  UK [62] | Small group lecture with student led case discussions/ activities; Student design session and delivered PH workshop in local school using presentations, activities, games  HP involved: doctors, dietitians, clinical psychologists | 11 h over 6 wk  N=15, final y MS | Pre/post survey; post intervention MCQ, focus group post intervention |
| Geller (2018)  USA [63] | Watch, discuss video clips of TV show depicting negative weight bias; small group discussions with facilitators; faculty member with bioethics expertise shares own experiences with weight to model self-disclosure  HP involved: faculty, physicians, residents | 90 min  N=6, 1^st^ y MS | Pre/post survey, 4 mon later |
| Cohen (2019)  USA [64] | MS randomized to read article on: genetic/ behavioral causes of overweight/ chronic headache; virtual patient encounter in clinical environment (counseling patients with obesity)  HP involved: NR | Duration: NR  N=119, 3^rd^/4^th^ y MS, 1^st^ y Rd, 52.1% F | Closed- ended content, analytic approach |
| Leedham-Green (2019)  Greece [65] | Interactive lectures; hand-outs; peer role-play, feedback; clinical opportunities, tutor support, MS conduct interventions with PWO; skills sheet facilitates consultation, goal setting, action-planning; patient handout facilitates diet advice  HP involved: GP | 4 wk  N=329 final y MS | Reflective learning essay thematically analyzed |
| Ockene (2021)  USA [66] | Web-based course; role-play; web-patient encounter, feedback, and enhanced clerkship experience with WMC-trained preceptors; counseling focused on the 5As and patient-centeredness  HP involved: Preceptors from FM/ IM clerkships, trained in WM | 4 h web based, 1^st^, 2^nd^/3^rd^ MS  N=629 MME, 687 traditional | OSCE; self-reported skills |
| Eichenberg (2023)  USA [67] | Didactic lecture, recorded and assigned to students in advance of small-group sessions; interactive small-group discussion on weight bias led by facilitator; case-based dialogue; student materials  HP involved: faculty/ residents from multiple specialties | 3 h workshop  N=353, 2^nd^ y MS | Post online quiz (short-essay questions/ MCQ); Post survey |
| Grunvald (2023)  USA [68] | MS into 2 groups: one exposed to SP (interview SP, use MI techniques for behavior changes, with feedback), other exposed to SP + interactive lecture (presentation and 4 real patients treated with interventions, discussions); role play with feedback from faculty/ peers; small group meetings  HP involved: faculty | 4 h sessions/ mon  N: SP group=72, 45.8% F, IL+SP=46, 41.3% F | Pre/post surveys |
| Renold (2023)  Switzerland [69] | Structured multi-dimensional semester course: interactive lectures; live surgery transmission; Gamification task with bariatric weight suits to experience different social situations in everyday life  HP involved: NR | 32 h lectures over 8 wk, 79, 3^rd^/4^th^ MS, 60.8% F | Pre/post questionnaire |
| Trofymenko 2024 USA [70] | Interactive web course using case-based learning, panel of patients undergoing treatment for obesity  HP involved: faculty | 2.5 h  N=103, 1^st^ y MS, 53% F | Pre/post questionnaire |

*BMI* body mass index, *C* control, *E* experimental, *EBM* evidence‐based medicine, *FM* family medicine, *F* female, *GP* general practitioner, *h* hour, *HP* Health professional/s, *HS* high school, *IL* interactive lecture, *IM* internal medicine, *MCQ* multiple-choice questions, *MI* Motivational Interviewing, *min* minute, *mon* months, *MS* medical students, *N* number, *NR* Not reported, not able to retrieve info from the study, *ob/gyn* obstetrics and gynaecology, *PA* physical activity, *PBL* problem-based learning, *PWO* patient with obesity, *SP* standardized patient, *wk* weeks, *WM* weight management, *WMC* weight management counseling, *WC* waist circumference, *y* year

**Supplementary Table 3** Additional characteristics of obesity education interventions in residency programs worldwide

| **Study** | **Teaching/ Learning methods, HP involved** | **Duration/ TA** | **Evaluation Tool/s** |
| --- | --- | --- | --- |
| Gonzalez (2006)  USA [71] | Pre-lecture orientation; written lecture; web based interactive module assigned readings; pediatric obesity clinics with nutritionist; small group meeting, PBL case discussion; nutritionist observe/ assess during counseling  HP involved: pediatric nutritionist | 20 min lecture, 1 h case-based discussion, 2 half d slots, N=6 PGY-2 Ped Rd | Pre/ post survey (immediately after, end of 3^rd^ y); Pre/ post MCQ |
| Huang (2009)  USA [72] | Multi resource web-based modules, power-point; supplementary materials, selected resources “pop-up” applications; role-play scenarios; case-based learning; training handouts, toolkit materials  HP involved: lecturer | Twice/ wk for 5 wk in outpatient rotation, N=24 Rd (Ped, Ped dental, PM | Feedback; Write-in boxes; quizzes after lectures |
| Burton (2010)  USA [73] | Interactive workshop; didactic lecture; audio-taped clinical encounters; MI practice with SP; role-playing with peer.  HP involved: NR | 3 h/ mon  N=77 IM/ Ped Rd, PGY 1-3, 54.4% F | Pre/post-questionnaire; post workshop evaluation |
| Laiteerapong (2011)  USA [75] | Chart review/audit; educational handout; case-based lecture; plan-do-study-act cycles.  HP involved: NR | Over 1 y  N=10, 2^nd^ y IM Rd | Documentation rates at baseline, 2 wk, 6 mon, 1 y post-intervention |
| Stahl (2011)  USA [74] | Online training program; clinic-based behavioral change intervention using behavioral planning sheet; flyer for community-based education; videos of clinic-based interventions (with teen, with younger child and parent)  HP involved: NR | 60 min  N=113 Ped Rd | Pre/posttests; patient/ parent follow-up interviews 4 wks after clinic visit |
| Wislo (2013)  USA [76] | Standardized training; brief PowerPoint; flashcards, games  HP involved: residents, and mentors | 1 h  N=57 FM Rd, 57% female | Pre-/ post surveys |
| Jay (2013)  USA [77] | Multiple interactive teaching modalities: didactic lectures, role playing, standardized patients, videotape review of patient encounter  HP involved: NR | 5 h, 3 weekly sessions  N=23 PC/ IM Rd in C/ intervention | Follow-up reviews of patient chart up to 1 y after index visit |
| Acosta (2014)  USA [78] | Multidisciplinary didactic sessions curriculum  HP involved: multidisciplinary team of academic obesity medicine lecturers, endocrinologist, psychiatrist, PH physician, nutritionist, physical therapist, bariatric surgeon, associate program director | 4 h, every 2 wk over 8-wk  N=75 IM 1^st^, 2^nd^, 3^rd^ y Rd, 49% F | Pre/post surveys (2 wk pre- /6 mon post): chart reviews of patients to assess clinical performance pre- and 6 mon after; patient-specific clinical outcome measure |
| Ren (2016)  USA [79] | 2 didactic presentations, 2 wks apart  HP involved: NR | NR, N=139 IM Rd, PGY1-3, 48% F | Pre/post medical record review; continuity clinic to assess effect of intervention |
| Iyer (2018)  USA [80] | Didactic session; case-based discussion; review sentinel articles; facilitated group discussion; facilitated group role play with feedback; simulated patient scenario with feedback.  HP involved: faculty | 3 h, 4 times over 6 mon  N=28 Primary Care/ Social IM Rd | Pre/ post survey 1 wk prior, 1 mon after curriculum |
| Carter (2019)  USA [81] | Didactic session; interactive question/ answer case-based scenarios, tutorial on EMR for obesity detection and follow-up once identified  HP involved: residents and attending physicians | 15 min  N=35 Rd | Pre/ post surveys: 1 wk prior, 9 mon after) and pre-post booster surveys; Patient chart review |
| Khandalavala (2020)  USA [82] | 5 Interactive web-based learning modules; grand rounds discussion; obesity-simulation empathy suit; HP involved: obesity medicine specialist, clinical psychologist, pharmacist, PH professor | Half day  N=28 FM Rd | Pre/ post surveys (immediate, 15 mon later) |
| Luig (2020)  USA [83] | Interactive lectures; empathy suit experience with reflection in small groups with expert preceptors; SP interviews: Rd debrief in small groups with preceptor, SP, peers; in-clinic patient practice of acquired skills.  HP involved: NR | Online, 8–11 h over 2 d  N=42 1^st^ y FM Rd, 57.1% female | Pre/ post survey; Qualitative narrative reflections, thematically analyzed |
| Faro (2022)  USA [84] | 5 vignettes via video-based communication assessment; diverse actors portray vignettes, videotaped them  HP involved: NR | NR  N=16 IM Rd | Analog patients rated Rd; debriefing interviews with Rd, thematically analyzed |
| Koran-Scholl (2023)  USA [85] | Didactic web-based module; video of 5 clinical vignettes.  HP involved: obesity medicine specialist, clinical psychologist, pharmacist, PH professor | 1 h  N=83 FM Rd | Pre/ post surveys: post-survey included open-ended qualitative questions to evaluate module |

*EMR* electronic medical records, *FM* family medicine, *h* hour, *HP* Health professional/s, *IM* internal medicine, *MCQ* multiple-choice questions, *mon* months, *N* number, *NR* Not reported, not able to retrieve info from the study, *PBL* problem-based learning, *Ped* pediatrics, *PGY* post graduate year, *PH* public health, *PWO* patient with obesity, *Rd* residents *SP* standardized patient, *wk* weeks, *y* year
